# Raising the Bar: Age‐adjust NT‐proBNP to improve specificity and reduce delay

**DOI:** 10.1002/ehf2.15427

**Published:** 2025-09-16

**Authors:** Lisa J. Anderson

**Affiliations:** ^1^ City St George's, University of London London UK

Based on population and reported mean incident rates,^1^ more than 2 million people are diagnosed with heart failure (HF) each year in Europe, and many more present with similar common signs and symptoms, requiring investigation and exclusion of HF. Demand for diagnostic services is outstripping supply and the problem is set to grow as crude HF incidence rises due to aging populations. Natriuretic peptides have a vital role to play as a triage tool in primary care, limiting the number of patients requiring onward referral for echocardiogram and specialist review, protecting resources for those most in need. The current single threshold of NT‐proBNP <125 pg/mL to rule out HF recommended in European and American Guidelines^2^, ^3^ has excellent negative predictive value but poor specificity, particularly in the elderly where HF burden is greatest.

Community presentation with HF symptoms and elevated NT‐proBNP represents a sentinel event, after which hospitalisations and mortality rise sharply,^4^ and delayed HF diagnosis is a global issue. In the United Kingdom, nearly 80% of HF diagnoses were reported to occur during emergency admissions,^5^ highlighting persistent delays despite long‐established national guidelines. In the United States, low rates of NP and echocardiographic testing are reported, leading to diagnostic delays and increasing proportions of hospital diagnoses.^6^ Women, socially deprived groups, and those with multiple comorbidities experience the greatest delays.^7^ Low‐ and middle‐income countries bear an even higher burden, with delayed diagnosis contributing to higher mortality rates.^8^ Late diagnosis is consistently linked to worse outcomes, and those diagnosed during hospitalisation have higher mortality rates than those diagnosed earlier in primary care.^9^, ^10^, ^11^

Age‐related variation in natriuretic peptide levels has long been recognized^12^ and confirmed by early population‐based studies.^13^ In 2010, an international multicentre study demonstrated that age‐specific NT‐proBNP thresholds outperformed a single fixed threshold for the detection of systolic dysfunction in primary care.^14^ Large population studies show that NT‐proBNP values rise steeply with age and >75% of healthy individuals >80 years exceeded the 125 pg/mL NT‐proBNP threshold.^15^ The 2023 HFA‐ESC Consensus Statement on NT‐proBNP testing recommended the introduction of age‐related thresholds with HF considered ‘likely’ in the outpatient setting if NT‐proBNP ≥125 pg/mL for those aged <50 years, ≥250 pg/mL between 50 and 75 years, and ≥500 pg/mL over 75 years.^16^ However, these cut‐points were determined from the 95^th^ percentile of the Generation Scotland cohort rather than from a prospective clinical study. As NT‐proBNP falls with increasing BMI, the statement also suggested lowering NT‐proBNP age‐adjusted thresholds by 25%, 30% and 40% for BMI ranges 30–35 kg/m², 35–40 kg/m², and >40 kg/m².

In the current issue of ESC HF, Taylor et al report the diagnostic accuracy of the ESC‐HFA age‐related NT‐proBNP thresholds in a retrospective cohort. The study employed English primary care data between 2004 and 2018 from the Clinical Practice Research Datalink (CPRD) and linked to inpatient Hospital Episode Statistics (HES). The CPRD archives hold data from around 15% of general practices in the UK and the large cohort undergoing NT‐proBNP testing (*n* = 155 347) enabled the assessment of age‐adjusted thresholds overall, in both sexes, and by body mass index (BMI). Patients entered the cohort on the date of their NT‐proBNP test and exited the cohort on the date of their HF diagnosis or after 6 months, whichever occurred first. HF diagnosis was obtained from diagnostic codes entered in primary care or during hospital admission or in cases where echocardiography findings were consistent with HF. The current ESC single threshold of ≥125 pg/mL showed a sensitivity of 95% and specificity of only 50%. Age‐adjusted thresholds for <50 years, 50–74 years, and ≥75 years had increased specificity (78%, 68% and 64%) but reduced sensitivity (84%, 89%, 84%) meaning some lower‐risk HF cases may initially be missed in the community. The negative predictive value was 95% or above across all age categories. For all age‐adjusted thresholds, sensitivity decreased with increasing obesity category. By lowering the NT‐proBNP age‐adjusted thresholds by 25%, 30% and 40% for increasing obesity categories as suggested in the ESC‐HFA statement, the sensitivity and specificity were comparable with the test performance seen in the cohort overall.

Large‐scale healthy European and U.S. cohorts show that NT‐proBNP levels are higher in women than men up to age 50, but lower in women compared with men after age 75.^15^, ^17^ Consistent with these population trends, Taylor et al found that age‐related thresholds demonstrated lower specificity in women than men under 50 years and higher specificity in women than men for those aged 75 years and older. When all age categories are analysed together, the age‐adjusted NT‐proBNP thresholds demonstrated comparable overall performance in men and women.

By applying higher thresholds for older groups (e.g., ≥500 pg/mL for those aged ≥75), the natural rise of natriuretic peptides with age is accounted for, enhancing test accuracy in older populations where prevalence is highest. This study demonstrated that while the ESC HFA age‐adjusted NT‐proBNP thresholds preserved reasonable overall sensitivity (87%), they increased overall specificity to 70%, compared with 50% with the single 125 pg/mL threshold. The total number of patients exceeding the age‐adjusted thresholds was reduced by one‐third compared with the single threshold, with the potential to substantially reduce the demand for echocardiograms and specialist referral.

The study offers large‐scale, retrospective, real‐world evidence from primary care, supporting the clinical applicability of the ESC HFA age‐adjusted NT‐proBNP thresholds. However, important limitations exist. Primary care coding can be unreliable, frequently depending on diagnoses made by non‐specialists in secondary care in the absence of echocardiography confirmation. While some coded as having HF may not have the condition, there are many other HF patients with incomplete or missing codes in GP records. The study was conducted in England, where national guidelines recommend further testing only if NT‐proBNP levels are over 400 pg/mL,^18^ potentially overlooking HF diagnoses with NT‐proBNP between 125 and 400 pg/mL. Additionally, the six‐month follow‐up window after NT‐proBNP testing—intended to capture hospital presentations or community deterioration—may be insufficient to identify all cases due to acknowledged diagnostic delays.

Efficient, accurate and timely HF detection remains a significant challenge, especially in community and primary care settings. Although the current NT‐proBNP threshold of 125 pg/mL provides excellent negative predictive value, its use as a trigger for further testing to avoid missing even mild HF cases is overly cautious and generates excessive referrals, thereby inadvertently compromising patient safety by overburdening echocardiography and specialist services. As populations age, the mismatch between the numbers of elderly people with common, non‐specific symptoms and the resources required to cope with the diagnostic demand is set to grow further. Delayed HF diagnosis is a global problem that worsens mortality, prolongs untreated symptoms and drives up healthcare costs. Rapid diagnostic pathways using more specific measures—like age‐adjusted NT‐proBNP thresholds—are essential to reduce delay, decrease unnecessary admissions and enhance care for high‐risk groups.

However, implementing age‐adjusted thresholds adds complexity to interpretation in primary care, whereas single thresholds are simpler to recall for general practitioners dealing with a multitude of different conditions.

Nephrology once faced the same challenges with serum creatinine—an imprecise biomarker affected by age, sex and other factors. Step‐wise global consensus building eventually led to the adoption of an age‐ and sex‐adjusted eGFR as a universal reporting standard. The same could be possible for NT‐proBNP—universal, equitable and clinically meaningful results at the point of reporting.

Globally billions are invested annually by combined public, charitable, pharmaceutical and device sectors in HF research, yet consensus on adjusted NP cut‐offs remains lacking after 25 years. Besides age, factors such as sex, BMI, race, ethnicity,^19^ and comorbidities affect NP levels. Prospective international studies with diverse populations and rigorous case adjudication are needed to establish optimal adjustments and implementation strategies.

With millions of new HF cases and rising crude incidence in aging populations, improved detection methods are urgently needed. Most HF research targets therapeutics and devices, while diagnostic pathways struggle to meet demand, meaning that many patients will never access available therapies. Investing in detection and implementation science will enable timely treatment delivery, reduce healthcare burdens and improve patient outcomes.

## Conflict of interest

Dr Anderson declares speaker fees from Espace, Radcliffe Education, SCIARC and Alnylam, Advisory board with Pharmacosmos, expert advisory work for National Institute of Clinical and Health Excellence (NICE), and travel and accommodation to lecture from Abbott and Roche. In addition, Dr Anderson is chair of the British Society for Heart Failure and chair of the National Health Service England Expert Advisory Group.
